# Supplementary material for: MetaRibo-Seq measures translation in microbiomes
Source: Nat Commun. 2020 Jun 29;11:3268. doi: 10.1038/s41467-020-17081-z (PMC7324362; doi:10.1038/s41467-020-17081-z)
Supplement: Supplementary file 10 — Supplementary Data 7 [file 41467_2020_17081_MOESM10_ESM.zip › File2/Confidence_High_Taxonomy/160591_out.krona.html]

Javascript must be enabled to view this page.

members
magnitude
magnitudeUnassigned
count
unassigned
taxon
rank

160591\_out

4

4
2
superkingdom

4
976
phylum

class
200643
4

4
171549
order

family
4
815

4
816
genus

1
28116
species

SRS049896\_contig\_number\_23037

1841855
3
species

SRS016753\_contig\_number\_4167SRS075398\_contig\_number\_870SRS150029\_contig\_number\_17126
